# Supplementary material for: Diarrhea Is a Hallmark of Inflammation in Pediatric COVID-19
Source: Viruses. 2022 Dec 6;14(12):2723. doi: 10.3390/v14122723 (PMC9783993; doi:10.3390/v14122723)
Supplement: Supplementary file 1 [file viruses-14-02723-s001.zip › viruses-1996476-supplementary.pdf]

viruses

Table S1. Subgroup analysis of inflammatory markers (exclusion of asymptomatic cases).

	Children with Diarrhea (n = 77)	Children without Diarrhea (n = 316)	*p*
**CRP, mg/L mean (SD)**	27.0 (65.0)	13.0 (26.1)	.004
**PCT, ng/mL mean (SD)**	2.8 (7.4)	0.6 (2.8)	.006
**Ferritin, ng/mL mean (SD)**	650 (1470)	271 (651)	.045

Abbreviations: CRP, C-reactive protein; PCT, procalcitonin.

Table S2. Subgroup analysis of inflammatory markers (exclusion of asymptomatic and mild-symptomatic cases).

	Children with Diarrhea (n = 44)	Children without Diarrhea (n = 162)	*p*
**CRP, mg/L mean (SD)**	43.8 (82.1)	17.6 (32.3)	.002
**PCT, ng/mL mean (SD)**	2.1 (7.6)	0.7 (3.3)	.030
**Ferritin, ng/mL mean (SD)**	819 (1737)	234 (268)	.048

Abbreviations: CRP, C-reactive protein; PCT, procalcitonin.

Table S3. Reference values of biochemical parameters evaluated in the study.

Biochemical parameters	Reference values
CRP, mg/L	0 – 5
PCT, ng/mL	0 – 0.5
Ferritin, ng/mL	7 – 140
WBC (10^3 cells/μL)	4.5 - 11.0
Neutrophils (10^3 cells/μL)	1.8 - 7.0
Lymphocytes (10^3 cells/μL)	1.0 – 4.8
Platelet count (10^3 cells/μL)	150 – 450
D-Dimer, ng/mL	0 – 500
CK-MB, ng/ml	0 – 7.2
hs-cTn, pg/mL	0 – 34

Abbreviations: CK-MB, creatine kinase-myoglobin binding; CRP, C-reactive protein; hs-cTn, high-sensitivity cardiac troponin; n, number; PCT, procalcitonin; WBC, white blood cells

Table S4. Univariable analysis.

Factors	aOR	CI 95%	*P*
Age < 12 m	ref	ref	Ref
Age 1 – 4 y	0.863	0.447-1.666	.661
Age 5 – 11 y	1.416	0.753-2.662	.280
Age 12 – 17 y	1.102	0.484-2.510	.817
1st wave (ancestral)	ref	ref	Ref
2nd wave (alpha)	1.663	0.771-3.584	.194
3rd wave (delta)	1.511	0.672-3.401	.318
4th wave (omicron)	1.077	0.523-2.217	.841
Female gender	0.777	0.468-1.291	.330
Non Caucasian race	0.947	0.311-2.881	.923
Prematurity	1.960	0.672-5.716	.218
Comorbidities	0.851	0.511-1.418	.536
Immunosuppression	0.286	0.086-0.949	.041
Fever	0.752	0.430-1.316	.318
Vomiting	4.368	2.379-8.020	<.001
Abdominal pain	7.359	3.619-14.966	<.001
Poor feeding	1.561	0.787-3.098	.203
Cough	1.728	1.048-2.849	.032
Respiratory distrees	0.686	0.296-1.591	.380
Seizures	0.248	0.058-1.060	.060
Headache	2.438	0.939-6.334	.067
Antibiotics	0.648	0.393-1.070	.090
Parenteral antibiotics	1.223	0.625-2.394	.557
Coinfections	1.547	0.866-2.764	.140
Pathological chest X-ray	1.152	0.640-2.074	.637
Interstitial disease	1.131	0.603-2.121	.700
Lobar/GGO	1.195	0.570-2.506	.637
Pathological abdominal US	3.055	1.063-8.774	.038
Abdominal lymphadenopathy	2.267	0.460-11.177	.315
Mesenteric fat hyperechogenicity	3.309	1.129-9.695	.029
Peritoneal effusion	2.267	0.460-11.177	.315
Intestinal wall thickening	0.493	0.048-5.035	.551

Abbreviations: aOR, adjusted odds ratio; CI, confidence interval; GGO, ground-glass opacities; US, ultrasounds.
